# Retroviral Insertion Polymorphism (RIP) of Porcine Endogenous Retroviruses (PERVs) in Pig Genomes

**DOI:** 10.3390/ani14040621

**Published:** 2024-02-15

**Authors:** Zhanyu Du, Cai Chen, Yao Zheng, Xiaoyan Wang, Chengyi Song

**Affiliations:** 1 College of Animal Science and Technology, Yangzhou University, Yangzhou 225009, China; dx120190120@yzu.edu.cn (Z.D.); 007302@yzu.edu.cn (C.C.); mz120180996@yzu.edu.cn (Y.Z.); wxyan@yzu.edu.cn (X.W.); 2 College of Grassland Resources, Institute of Qinghai-Tibetan Plateau, Southwest Minzu University, Chengdu 610225, China

**Keywords:** pig, retrotransposon, full-length ERV polymorphism, growth performance

## Abstract

**Simple Summary:**

Retrotransposons in pigs account for approximately 37.13% of the genome, while long terminal repeats (LTRs) make up 7.56%. These LTR retrotransposons consist of two identical sequences that flank an internal protein-coding region, known as an endogenous retrovirus (ERV). When an insertion polymorphism integrates into a genomic region that harbors functional ERV elements, such as promoters, enhancers, and insulators, it may contribute to phenotypic variation. In this study, we explored the insertion polymorphisms generated by the mobilization of full-length ERVs (Fl-ERVs). Through comparative genomic analysis, we identified 18 insertion polymorphic sites that were generated by Fl-ERVs, and evaluated the genetic impact of one Fl-ERV insertion polymorphism in the STAB2-like gene.

**Abstract:**

Endogenous retroviruses (ERVs) are one of the superfamilies of long terminal repeat retrotransposons (LTRs) in mice and humans. Approximately 8% of the pig genome is composed of sequences derived from LTRs. While the majority of ERVs in pigs have decayed, a small number of full-length copies can still mobilize within the genome. This study investigated the unexplored retroviral insertion polymorphisms (RIPs) generated by the mobilization of full-length ERVs (Fl-ERVs), and evaluated their impact on phenotypic variation to gain insights into the biological role of Fl-ERVs in pigs. Overall, 39 RIPs (insertions or deletions relative to the pig reference genome) generated by Fl-ERVs were predicted by comparative genomic analysis, and 18 of them were confirmed by PCR detection. Four RIP sites (D5, D14, D15, and D18) were further evaluated by population analysis, and all of them displayed polymorphisms in multiple breeds. The RIP site of ERV-D14, which is a Fl-ERV inserted in the STAB2-like gene, was further confirmed by sequencing. Population analysis of the polymorphic site of ERV-D14 reveals that it presents moderate polymorphism information in the Large White pig breed, and the association analysis reveals that the RIP of ERV-D14 is associated with age variations at 30 kg body weight (*p* < 0.05) and 100 kg body weight (*p* < 0.01) in the population of Large White pigs (*N* = 480). Furthermore, the ERV-D14 RIP is associated with changes in the expression of the target gene STAB2-like in the liver, backfat, and leaf fat in Sushan pigs. These data suggest that some Fl-ERVs are still mobilizing in the pig’s genome, and contribute to genomic and phenotypic variations.

## 1. Introduction

Transposable elements (TEs) are mobile components found in eukaryotic genomes that directly impact their size, structure, and function. The presence and distribution of TEs in eukaryotic genomes play a fundamental role in the process of evolution, as TEs are a significant source of new genetic material [1]. Approximately 40% of mammalian genomes are derived from TEs, which are composed of DNA transposons (1–2%) and retrotransposons (around 40%) [2]. DNA transposons integrate into the host genome through a “cut and paste” mechanism, while retrotransposons expand by employing a “copy and paste” technique [3].

Retrotransposons can be classified into two main groups: non-LTR retrotransposons, including LINEs and SINEs, and LTR retrotransposons. The latter consists of two identical LTRs flanking an internal protein-coding region called an endogenous retrovirus (ERV). It is common for LTR retrotransposons to undergo homologous recombination, resulting in the formation of solo-LTRs [4]. Endogenous retroviruses (ERVs) are one of the superfamilies of mouse and human LTR retrotransposons. Porcine endogenous retroviruses (PERVs) show different copy numbers in different organs, indicating that they are active [5,6,7,8]. Moreover, ERVs do not lose their retrotransposition ability; this was also shown for other ERV [9], in a study that showed a frequency of retrotransposition of up to 1.2 × 10^−6^ for PERV and 5 × 10^−7^ for MoMLV per cell per generation in single experiments, indicating that a PERV retrotransposition happens more than twice as often as a MoMLV retrotransposition. Our previous findings indicate that ERV6A and ERV6B, which possess the gag, pol, and env domains, represent the most recently classified family with potential transposable activity [10].

Transposons act as functional reservoirs of gene regulatory networks, promoters, enhancers, repressors, insulators, and chromatin boundaries [11]. They are also reservoirs of non-coding RNAs and proteins. Evidence suggests that ancestral LTR retrotransposons and LINEs expressed proteins to mediate retrotransposition, but most of them underwent deleterious mutations and/or epigenetic silencing [12]. Despite the potential advantages of TEs, many insertions result in a disease state, or even a lethal allele. For instance, the SINE insertion in IDO2 had a negative impact on the reproductive traits of litter birth-weight, which was significantly (*p* < 0.05) affected in the Large White pig breed [13]. A recent study on the swamp buffalo (*Bubalus bubalis*) [14] reported that the 2809-bp-long LINE-1 insertion in the agouti signaling protein (*ASIP*) gene is the causative mutation for the white coat phenotype. A retroviral insertion in the tyrosinase (*TYR*) gene is associated with the recessive white plumage color in the Yeonsan Ogye chicken [15].

Over the course of evolution, mammalian genomes have accumulated numerous retrotransposed sequences. These sequences can be categorized into two primary groups: long terminal repeat (LTR) retrotransposons, which include ERVs, and non-LTR retrotransposons. Non-LTR retrotransposons further consist of long interspersed nuclear elements (LINEs) and short interspersed nuclear elements (SINEs) [16,17,18].

ERVs are defined as inheritable genetic elements that closely resemble the proviruses formed after an infection by an exogenous retrovirus [19,20]. They are ancient remnants of retroviral infections that have integrated into the germ line DNA and are inherited across generations. Despite their classification as genomic fossils, ERVs can significantly influence genome structure and function. ERVs can modulate gene expression, contribute to genetic variation and drive evolution. They have also been co-opted by the host for beneficial roles in some instances [21,22].

It is believed that PERVs are genetic remnants of ancient viral infections that have become integrated into the DNA of germ cells and are subsequently inherited in subsequent generations. In a prior investigation, we conducted a thorough annotation of transposable elements (TEs) within the pig genome and estimated their ages based on transposons. Within the pig genome, retrotransposons constitute approximately 37.13% (929.09 MB), with LINEs, LTRs, and SINEs accounting for 18.52%, 7.56%, and 11.05%, respectively. Among the retrotransposons, LINEs constitute 18% of the genome, making them the most abundant category in the pig genome [10]. Worth mentioning, young SINE elements in the pig genome play crucial roles in new genetic variations and the evolution of pigs [14]. LINEs and SINEs, despite lacking LTRs, also play crucial roles in shaping the genome. LINEs, which are typically longer sequences, have the ability to copy themselves and insert into new locations in the genome, a process that can lead to genetic variation and disease. SINEs, although shorter and lacking the ability to transpose themselves, can be mobilized by LINEs and also contribute to genetic diversity [19,23,24,25].

Eighteen distinct families have been identified for ERVs, ranging from ERV1 to ERV18 [10,26]. Despite the overall degradation of most ERVs, ERV6 has exhibited heightened activity in the last 10 million years. Numerous copies of ERV6 encode extended peptides that contain intact gag, pol, and env domains [10]. Expression profiles and promoter activities of recently formed retrotransposons, in both the sense and antisense directions, have been analyzed and characterized. Furthermore, the impact of these retrotransposons on long non-coding RNAs (lncRNAs) and protein-coding genes was assessed by mapping the mobilome landscapes at both the genomic and transcriptomic levels [27,28]. An evident bias in the distribution of retrotransposon composition, location, and orientation was noted in both lncRNAs and protein-coding genes, as well as their transcripts [29]. In this aspect, beyond the insertion of a 192 bp ERV fragment in the first intron of porcine TLR6, it also functions as an enhancer, correlating with the elevated expressions of TLR6 and TLR1 [30]. 

Pigs play a crucial role as a significant food source, contributing to approximately 40% of the global meat consumption. Additionally, pigs are highly valued as model organisms because of their striking anatomical, physiological, and genetic similarities to humans. This similarity renders pigs exceptionally valuable in the study of various human disorders, making them a valuable resource in research and experimental studies [31].

Over the past two decades, pigs have been recognized as a crucial mammalian model for studying human diseases and other biomedical applications, primarily due to their size and physiological parameters, which are comparable to those of humans. Compared to rodents, pigs share many significant similarities with humans in their cardiovascular, respiratory, endocrine, and immune systems [32,33]. Consequently, due to the extremely limited number of human donors available for transplantation, pigs have become increasingly important as potential organ donors in xenotransplantation [34].

However, the safety of the therapeutic use of porcine materials remains unclear due to the presence of a range of active retrotransposon families in the pig genome, particularly PERVs [35]. The ability of retrotransposons to re-infect or move within the genome gives them an inherent propensity to potentially affect host health and genomic stability. It has been demonstrated that two subfamilies of PERV (γ1A and γ1B) can infect human cells, and one (γ1C) can infect porcine cells [35,36]. Non-LTR retrotransposons have unequivocally been shown to pose a risk to genomic stability, as evidenced by more than 90 reported cases of de novo insertions of active LINE and SINE elements that are responsible for genetic disorders in humans [37], Two cases of gene function alterations associated with the insertion of L1 retrotransposons into the pig genome suggest that L1 elements in the porcine lineage are still active [38,39].

Despite retrotransposons being prevalent in mammalian genomes and biologically significant, only a limited number of pig retrotransposons have been investigated. The aim of this study was to establish a reference for pig breeding and human xenotransplantation. The study involved detecting ERVs across 19 pig genomes at a genome-wide scale. Multiple de novo pipelines were utilized to obtain full-length ERVs (Fl-ERVs). Polymorphisms of these Fl-ERVs in various pig breeds were identified, and their potential impact on the pig genome and phenotypic traits was assessed. Comprehending the role and impact of ERVs is crucial not only for pig breeding but also for research on human xenotransplantation. However, the existing research on pig ERVs remains limited despite their prevalence and biological significance, underscoring the necessity for further investigation in this field.

## 2. Materials and Methods

### 2.1. Ethical Statement

The collection of biological samples and experimental procedures involved in this study were approved by the Animal Experiment Ethics Committee of Yangzhou University (No. NSFC2020-dkxy-02, 27 March 2020).

### 2.2. Fl-ERV Annotation of Pig Genome

Eighteen assembled non-reference pig genomes were downloaded from the NCBI genome database, along with the reference genome (Sus scrofa 11.1, listed in Table S1). The RepeatMasker program (version 4.1.2, -nolow) was used for ERV annotation, utilizing the ERV sequences obtained in an earlier study [10]. ERVs encoding viral proteins (gag, pol, and env), flanked by detectable LTRs and exceeding a length of 5 kb, were designated as full-length ERVs (Fl-ERVs). These were extracted from the downloaded genomes based on the ERV annotation results. Genscan was used to translate the obtained Fl-ERVs, and the protein domains were annotated using the online HMMER program. PERVs are typically classified into three types, PERV-A, -B, or -C, based on env sequence differences. PERV-A and -C recombine to generate new PERV variants PERV-A/C, which infect human cells [40], but here we name them with ERV-I, ERV-II, and ERV-III, respectively.

### 2.3. Fl-ERV Polymorphism Identification

Ear tissues from commercial breeds (Duroc, Large White, Landrace) and Chinese native breeds (Meishan, Sujiang, Sushan, Rongchang, Ningxiang, Bama, Laiwu, Tibetan, and Banna) were sampled for the purpose of polymorphism detection through PCR analysis. Each breed was represented by three individuals. The DNA extraction from ear tissue was performed using the Takara DNA Extraction Kit (Treasure Biology Co., LTD, Dalian, China).

Subsequently, equal amounts of genomic DNA samples from three individual genomes per breed were pooled together and subjected to PCR analysis in order to detect the predicted ERV insertion polymorphisms. Considering that most Fl-ERVs have a length exceeding 8 kb, double PCR primer pairs were designed and utilized for genotype verification. The strategy employed for primer design is illustrated in Supplementary Figure S1A.

### 2.4. Genotyping Analysis

As the ERV-FL is more than 8000 bp in length within the genome, we have designed primers targeting the ERV-FL region. However, for sequence amplification purposes, we only allow enzyme activity in the PCR system to amplify a range of 1500–1800 bp. In the first round of amplification, if a band within this size range (1500–1800 bp) appears, it indicates two possible scenarios: either there is no homozygous ERV insertion or there exists a heterozygote genotype in the pig genome. Subsequently, we employ one side of an ERV-FL primer and a shorter LTR inside primer to amplify the same sequence. If a shorter band ranging from 700–1000 bp is observed, it signifies that this RIP genotype must be heterozygous (labeled as ERV^+/−^). Conversely, if no band is present, it suggests an absence of homozygous ERV insertion genotype (labeled as ERV^−/−^). On the contrary, in the first round, if no band is detected, there is a high probability of homozygous ERV insertion. If the LTR primer shows a band (700–1000 bp), it indicates homozygous ERV insertion and is labeled as ERV^+/+^. Our objective is to obtain enriched polymorphic types across 12 pig breeds by eliminating individuals that exhibit homozygous insertions, heterozygotes, or homozygous deletions within the entire breed population. The complete length of the ERV-D14 insertion was verified through TA cloning and subsequent sequencing, with the primer design illustrated in Supplementary Figure S1B.

### 2.5. Association Analysis of Polymorphic Sites with Population Genetics

The growth performance data for Large White pigs (*N* = 480), including the number of days to reach 30 kg and 100 kg, as well as backfat thickness, were gathered and adjusted. These data were then utilized for a correlation analysis with genotypes. Statistical analyses were performed using one-way ANOVA, followed by Tukey’s post hoc test, with the aid of SPSS 17.0 (SPSS, Chicago, IL, USA). The age at which the pigs reached a body weight of 100 kg was adjusted according to a formula recommended by the China National Swine Genetic Assessment Scheme. A *p*-value less than 0.05 indicates a significant difference, while a *p*-value less than 0.01 indicates an extremely significant difference. The Hardy–Weinberg equilibrium was assessed using the following formula: ${\left(x\right)}^{2}=\sum_{i=1}^{n}\frac{\left(\left|O_{i}-E_{i}\right|-\frac{1}{2}\right)}{E_{i}}\left(df=1\right)$; *O_i_* is the actual observed value; *E_i_* is the theoretical value; *n* is the number of alleles.

### 2.6. Expression Analysis of the ERV-D14 Insertion Targeted Genes

For the expression analysis, 48 Sushan pigs, each 6 months old, were slaughtered, and the ERV-D14 polymorphism in these pigs was genotyped by PCR. Subsequently, tissues, including the liver, longissimus dorsi, backfat, leaf fat, and leg muscle, were sampled for RNA extraction using Trisol solution. The cDNA was then transcribed using the Tiangen FastKing RT Kit (Tiangen Biochemical Technology, Beijing, China). The expression of the target gene was detected via qPCR for different genotypes, with GAPDH primers set as positive. The 2^−∆∆CT^ method was used to calculate the gene expression differences between the various genotypes. The differences were analyzed using a *t*-test with GraphPad software (Prism 9.0).

## 3. Results

### 3.1. ERV Re-Annotation in the Genomes of Pig

**Figure 1:**
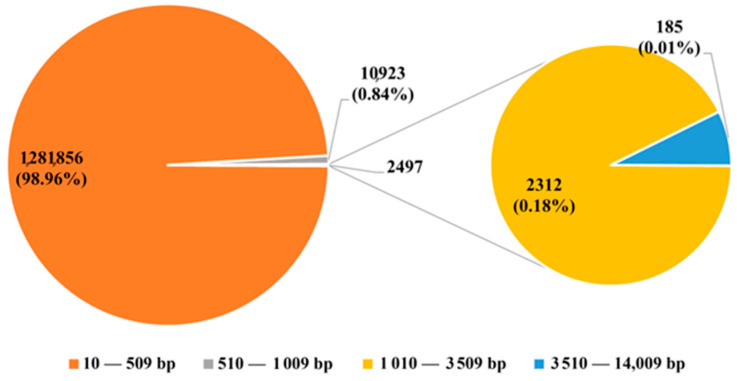
The number of ERV insertion fragments for different lengths by RepeatMasker.

The repeat library, which contains all identified ERV sequences previously published [7], was used to annotate the ERV distribution in 18 assembled non-reference genomes and 1 reference genome (Sscrofa11.1, GCF_000003025.6). The RepeatMasker annotation data reveal that the genomic coverage of ERVs across these genomes is approximately 6.94%, ranging from 6.42% to 7.14%, as summarized in Supplementary Figure S2. Most of these ERV sequences are derived from ERV-I elements (73.4%), followed by ERV-II (16.1%) and ERV-III (10.5%). Based on the ERV annotation using the well-assembled reference genome, about 99.8% of the ERV insertion fragments are less than 1 kb in length in the genome. Only 0.19% and 0.01% of ERV insertions are larger than 1.01 kb and 3.50 kb in length, respectively (Figure 1). This indicates that the vast majority of ERVs are truncated copies and are less than 1 kb in the reference genome.

### 3.2. Fl-ERV Insertion Polymorphism Detection and Correlation Analysis with Economic Traits

A total of 39 full-length ERV RIPs were predicted by comparing genomic coordinates, including 23 FL-ERV insertions and 16 deletions (relative to the Duroc reference genome), and all 39 full-length were annotated by the youngest activated ERV6A and ERV6B [7]. These are summarized in Table 1 and Supplementary Table S2. These RIPs were then evaluated by PCR using two pairs of primers with genomic DNA samples from 12 pig breeds, as described in the methods. Ultimately, 18 FL-ERV RIPs were confirmed by PCR (Table 1, Supplementary Figures S3 and S4), indicating that FL-ERV insertions are highly polymorphic. Four ERV-RIPs (ERV-D18, ERV-D14, ERV-D15, and ERV-D5) were further genotyped in a small population (24 individuals) of Large White pigs, and all showed polymorphism (as shown in Supplementary Figure S5). They were then genotyped in a large population (480 individuals) of Large White pigs, and their genotypes were found to be associated with economic traits. The ERV-D14 polymorphism is significantly (*p* < 0.01) correlated with the days required to reach 30 kg and 100 kg, as presented in Table 2. The ERV-D14 RIP was further confirmed by sequencing and a population genetic analysis was conducted in five other breeds. This RIP displays medium polymorphic information content (*PIC* = 0.26–0.37) in six pig breeds and conforms to the Hardy–Weinberg equilibrium (*p* < 0.05) (Table 3).

### 3.3. Impact of Fl-ERV RIP on the Gene Expression Analysis in Sushan Pigs

**Figure 2:**
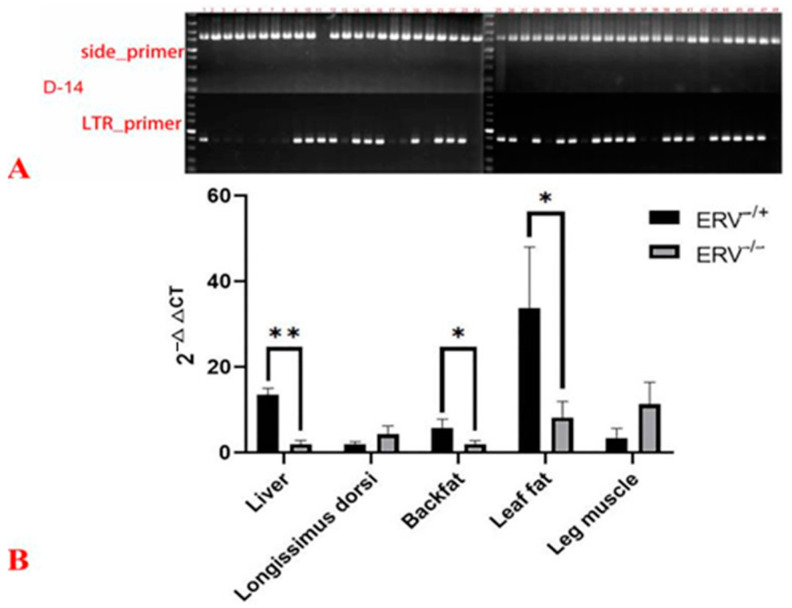
The result of genotyping and expression analysis of the target gene in Sushan pig breed. (**A**): The PCR genotyping for ERV-D14 insertion polymorphism in 48 Sushan pigs. (**B**): The expression analysis of STAB2-like gene for different genotypes. The detected tissue including liver, longissimus dorsi, backfat, leaf fat, and leg muscle of Sushan pigs. Significant difference (*p* < 0.05) was labeled with *, and extremely significant difference (*p* < 0.01) was labeled with **.

The flanking sequences (4 kb upstream and downstream) of the ERV-D14 insertion were extended and mapped to the reference genome for target gene annotation. We found that ERV-D14 is inserted in the 10th intron of the STAB2-like gene. To investigate the potential impact of the ERV-D14 insertion on the target gene, ERV-D14 was further genotyped in 48 Sushan individuals via PCR using side_primer and LTR_primer, and the genotyping results are shown in Figure 2A. From the electrophoretogram results, we found that 19 Sushan pig individuals presented the ERV^+/−^ genotype, 28 individuals were ERV^−/−^ genotype, and only 1 individual was ERV^+/+^. Considering the ERV-D14 insertion location in the 10th intron of the STAB2-like gene, we designed a primer pair spanning exon 7 and exon 8 to explore the effect of the insertion on target gene expression. The qPCR result of the ERV-D14 insertion reveals those animals with the ERV^+/−^ genotype display significantly higher expression of the target gene than those with the ERV^−/−^ genotype (*p* < 0.01) in the liver. Additionally, higher expression of the target gene in animals with ERV^+/−^ genotypes in backfat (*p* = 0.0167) and leaf fat (*p* = 0.0396) tissues was observed, compared with those of ERV^−/−^ genotype individuals (Figure 2B).

## 4. Discussion

The integration of retrotransposons can have a significant impact on gene activity and phenotype by affecting the regulatory elements, such as promoters and enhancers, of genes [41]. Phenotypic variations caused by the insertion of some ERVs have been observed in domesticated animals, including chickens [41], dogs [42], and cats [43]. In the pig genome, ERVs make up approximately 8% of its composition. Identifying polymorphisms resulting from ERV insertions in the domestic pig genome could improve the chances of discovering structural variations responsible for phenotypic variations, as compared to single nucleotide polymorphisms (SNPs). In particular, the presence of Fl-ERV insertion polymorphisms, which typically consist of large structural variations exceeding 5 kb, can result in a stronger genetic impact. In this study, we performed bioinformatic analysis on 19 assembled pig genomes, from which we obtained 39 Fl-ERV insertion polymorphic sites. Subsequently, we confirmed that 46.15% (18 out of 39) of these Fl-ERV insertions were indeed polymorphic through PCR detection. This finding indicates that certain full-length ERVs still retain the capability to mobilize and influence the pig genomes, some of which may contribute to phenotypic variations and population differentiation, despite the majority of ERVs in pig genomes being considered as fossils.

It is worth mentioning that the examination of multiple tissues would provide valuable insights into the variability of PERV copy numbers and their activity in different organs [5,6,7,8]. We also find the topic “the copy number of proviruses is different in different tissues of a single pig and even increases with age” to be very interesting and compelling. This phenomenon is closely related to the activity of ERVs in somatic tissues and cells. Active ERVs can undergo transposition events in different cells and tissues at different stages, leading to differences in copy numbers. Consequently, accurately measuring these copy numbers can be challenging. In the present study, the predicted retrotransposition insertion points (RIPs) were based on the assembled genomes obtained through sequencing. These genomes were assembled using DNA sequences derived from multiple tissues. Furthermore, a larger number of animals were used in the assembly process. As a result, the final RIPs obtained from the assembled sequences represent germline mutations that are heritable, rather than instabilities in somatic cells. Our data, which demonstrate the presence of RIPs across different breeds and animals, strongly suggest that retrotranspositions of ERVs occur in germline cells.

Additionally, we discovered that ERV-D14 is embedded within an intron of the STAB2-like gene, and the ERV-D14 insertion polymorphism exhibited a correlation with growth rate variations in Large White pigs. qPCR analysis shows that the ERV-D14 insertion could potentially alter the expression of the targeted gene in various tissues, such as liver, backfat, and leaf fat, in Sushan pigs. Previous research has highlighted the involvement of the STAB2 locus in hyaluronic acid (HA) metabolism, which includes HA synthases, hyaluronidases, and other receptors [44], as well as immunogenic activities [45]. However, the exact biological function of the STAB2-like gene remains largely unknown. Our results suggest that the STAB2-like gene might play a role in regulating animal growth through an unidentified mechanism, which merits further investigation. Furthermore, additional research is needed to establish the causal relationship between the ERV-D14 insertion polymorphism and the observed phenotypic effects.

## 5. Conclusions

In total, 18 polymorphic insertions of full-length ERVs (Fl-ERVs) were identified in the pig genome. The study reveals that Fl-ERV insertions in pig genomes are highly polymorphic and are annotated by the youngest activated ERV6A and ERV6B, indicating that some full-length ERVs are still active in transposition and continue to play roles in shaping the evolution of the pig genome. Some of them may contribute to phenotypic variation and population differentiation, even though most ERVs in pig genomes are fossils. The ERV-D14 insertion polymorphism was found to be associated with growth traits and the expression levels of the STAB2-like target gene. This ERV-D14 retrotransposon insertion polymorphism (RIP) may serve as an important molecular marker to assist in pig breeding and provide a foundation for research in pig genome pre-mining efforts.

## Figures and Tables

**Table 1:** The predicted polymorphic insertions of Fl-ERV in the genomes of pig.

Name	Insertion/Deletion	Strand	Chr	ERV_start	ERV_end	gag_aa Length	pol_aa Length	env_aa Length
D2	NS_3_D;CB_2_D	+	chr8	51,570,546	51,579,460	471	690	220
D3		+	chr5	92,185,134	92,194,051	417	881	220
D5	NS_2_D	+	chr1	132,020,281	132,028,360	271	767	141
D9	BM_3_D; MSBJ_7_D; NS_10_D; NX_6_D; CB_4_D	+	chr17	3,793,914	3,802,815	430	1088	468
D10		+	chrUn_NW_018085136v1	563,793	572,725	275	911	394
D12	NS_8_D	+	chr3	51,108,602	51,117,361	492	901	121
D14		+	chrUn_NW_018085331v1	13,836	22,597	492	979	192
D15	KY_3_D; NH_2_D	+	chr14	119,667,621	119,676,299	457	706	135
D17	NS_7_D	+	chr8	15,319,428	15,328,187	492	559	122
D18		+	chr16	59,571,885	59,580,646	492	876	192
D21	NS_5_D;CB_7_D	+	chr9	138,895,584	138,904,340	492	979	192
BM_2_D	Deletion	+	Chrx	66,738,746	66,744,246	492	743	156
MSBJ_1_D	Deletion	+	chr7	126,674,088	126,679,601	417	1032	141
MSBJ_8_D	Deletion	C	chr9	62,631,695	62,637,198	492	979	192
MSBJ_3_D	Deletion	C	chr1	250,447,148	250,452,649	492	889	121
RC_1_D	Deletion	C	LUXR01088996.1	1,997,965	2,007,394	515	960	631
NX_2_D	Deletion	+	chr2	151,052,074	151,053,575	492	979	192
NX_5_D	Deletion	C	chr9	24,240,887	24,242,393	497	1004	198

Note: BM: Bama pig; CB: Cross-breed; NX: Ningxiang pig; NS: Sicilian pig; MSBJ: Meishan Pig; RC: Rongchang Pig; D: Duroc pig; KY: Kenyan pig. Strand C means complementary strand.

**Table 2:** Correlation analysis of ERV-D14 insertion polymorphism with economic traits in Large White pigs.

RIP Name	Genotype	30 kg/Days	100 kg/Days	BTW
ERV-D14	ERV^−/−^ (*N* = 74)	76.89 ± 7.68 A	164.46 ± 7.08 a	10.62 ± 2.58
	ERV^+/+^ (*N* = 133)	73.59 ± 9.47 B	161.19 ± 6.48 b	11.11 ± 2.59
	ERV^+/−^ (*N* = 272)	74.11 ± 8.35 B	160.97 ± 6.94 b	11.47 ± 9.24

Note: The same letter indicates no significant difference; different lowercase letters represent extremely significant difference, *p* < 0.01, different capital letters represent significant difference, *p* < 0.05; 30 kg/days: the age (unit: days) for reaching the weight of 30 kg; 100 kg/days: the age of reaching 100 kg weight (unit: days); BTW: corrected backfat thickness of 100 kg body weight (unit: mm).

**Table 3:** Population genetics analysis of ERV-D14 insertion polymorphism in 6 pig breeds.

Breeds	Count	Genotype Frequency (%)			Allele Frequency (%)		Hardy–Weinberg	Equilibrium	PIC
		+/−	−/−	+/+	+	−	*X* ^2^	*p*	
Large White	24	62.50	0.00	37.50	68.75	31.25	4.96	0.06	0.34
Duroc	24	37.50	0.00	62.50	81.25	18.75	1.28	0.26	0.26
Mi	24	45.83	54.17	0.00	22.92	77.08	2.12	0.15	0.29
Sujiang	24	41.67	58.33	0.00	20.83	79.17	1.66	0.20	0.28
Sushan	24	54.17	45.83	0.00	27.08	72.92	3.31	0.07	0.32
Landrace	24	25.00	41.67	33.33	45.83	54.17	5.92	0.02	0.37

Notes: ERV^+/+^, ERV insertion homozygous genotype; ERV^+/–^, ERV insertion heterozygous genotype; ERV^–/–^, No ERV insertion homozygous genotype; ERV^+^, ERV insertion allele; ERV^–^, no ERV insertion allele; PIC, polymorphic information content.

## Data Availability

All data needed to evaluate the conclusions in this paper are present either in the main text or the supporting information.

## Supplementary Materials

The following supporting information can be downloaded at: https://www.mdpi.com/article/10.3390/ani14040621/s1, Supplementary Figure S1. Design principles of Fl-ERV polymorphic primers and TA clone primers; Supplementary Figure S2. Proportion of ERV in the total number of retrotransposons in 19 pig breeds; Supplementary Figure S3. Polymorphism identification of Fl-ERV in 12 pig breeds; Supplementary Figure S4. Polymorphic heat maps of Fl-ERV in 12 varieties; Supplementary Figure S5. Identification of 5 Fl-ERVs in 12 pig breeds; Supplementary Figure S6. Polymorphism identification of ERV-D14 locus in 480 Large White pigs; Supplementary Figure S7. Analysis results of association between ERV-D14 and histone data; Supplementary Figure S8. Amplification of ERV-D14 in the intercalated Sushan pig genome; Supplementary Figure S9. TA clone results of ERV-D14 and compare with reference breed of Duroc; Supplementary Table S1. Details concerning the genomes of the 20 pig breeds investigated in this study; Supplementary Table S2. Fl-ERV prediction of 19 pig breeds.
