# Supplementary figures and images for: Retroviral Insertion Polymorphism (RIP) of Porcine Endogenous Retroviruses (PERVs) in Pig Genomes
Source: Animals (Basel). 2024 Feb 15;14(4):621. doi: 10.3390/ani14040621 (PMC10886097; doi:10.3390/ani14040621)

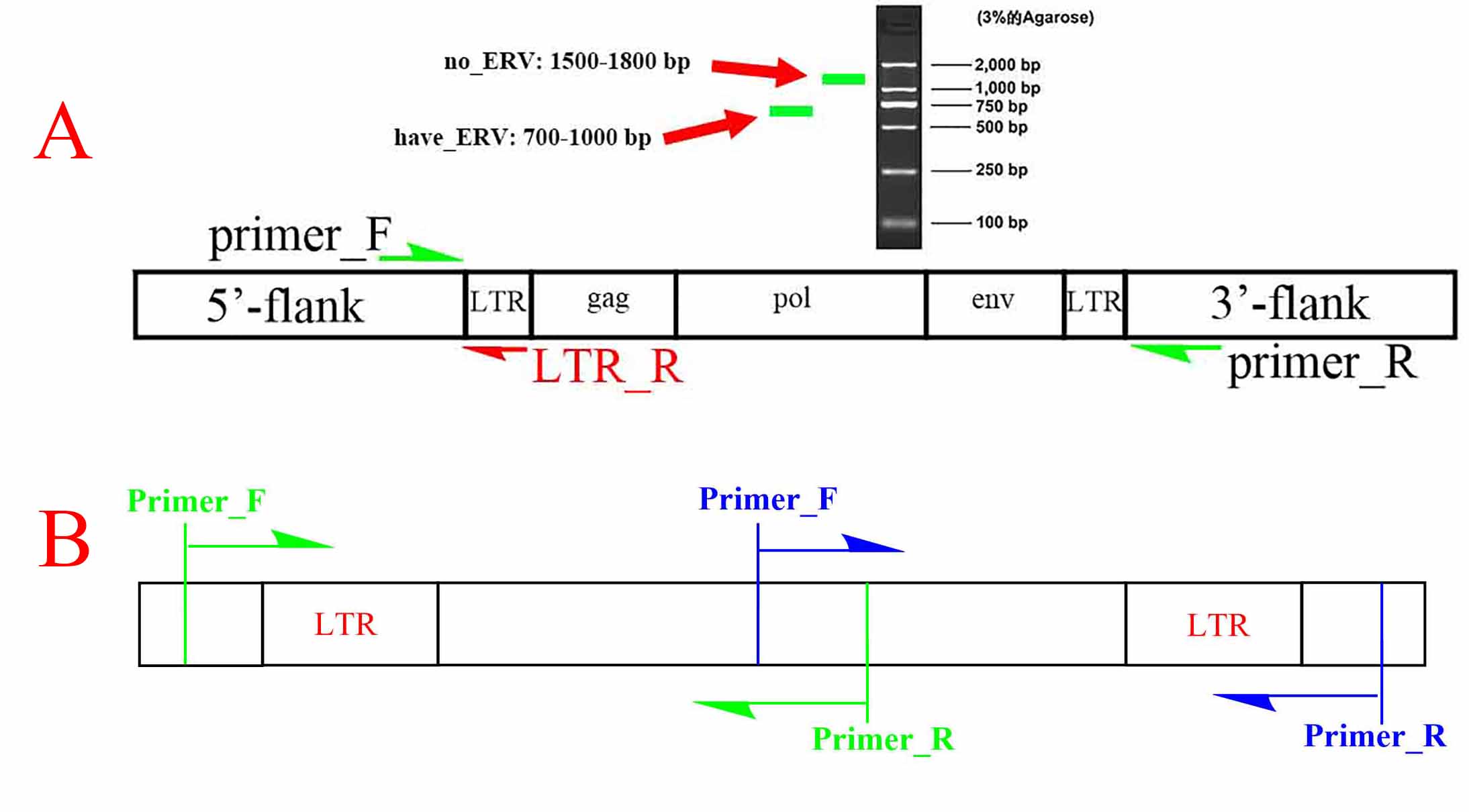

Supplement: Supplementary file 1 [file animals-14-00621-s001.zip › Supplementary Figure S1. Design principles of Fl-ERV polymorphic primers and TA clone primers.jpg]

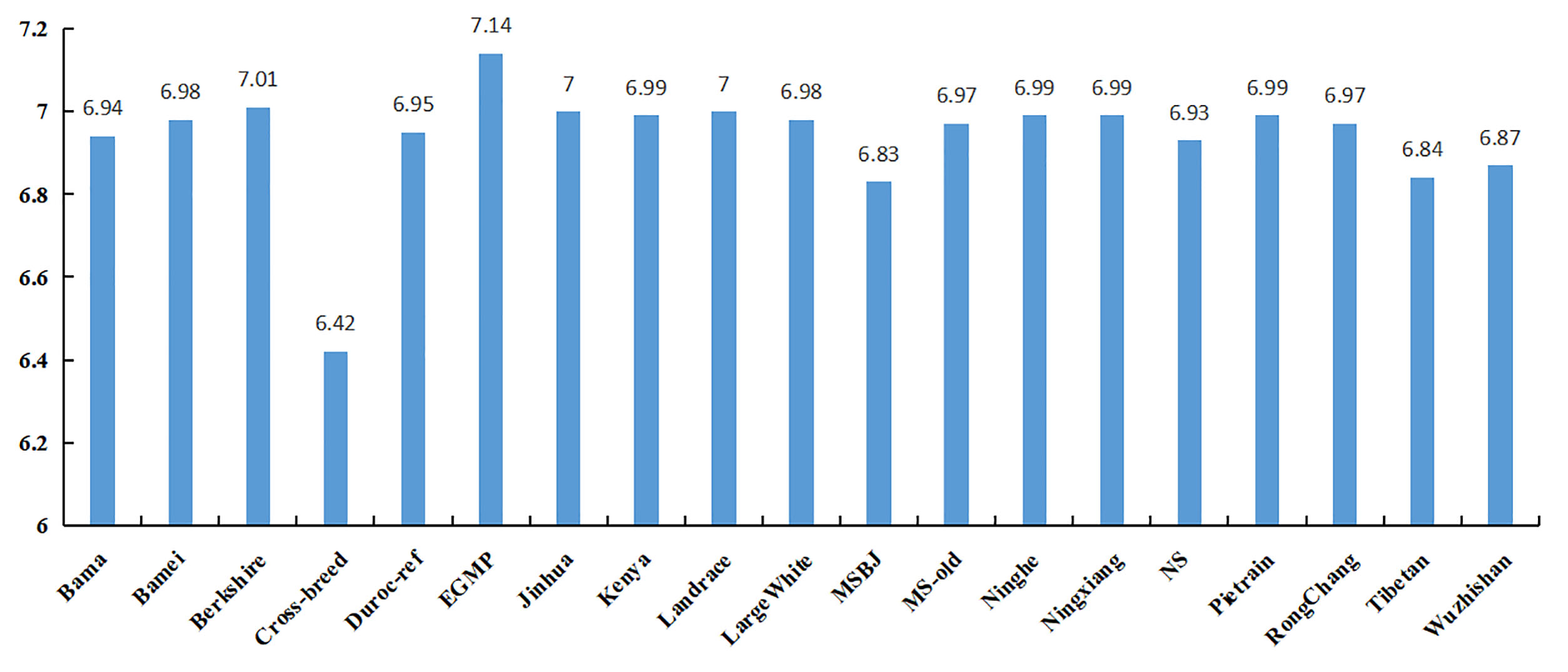

Supplement: Supplementary file 1 [file animals-14-00621-s001.zip › Supplementary Figure S2. Proportion of ERV in the total number of retrotransposons in 19 pig breeds.jpg]

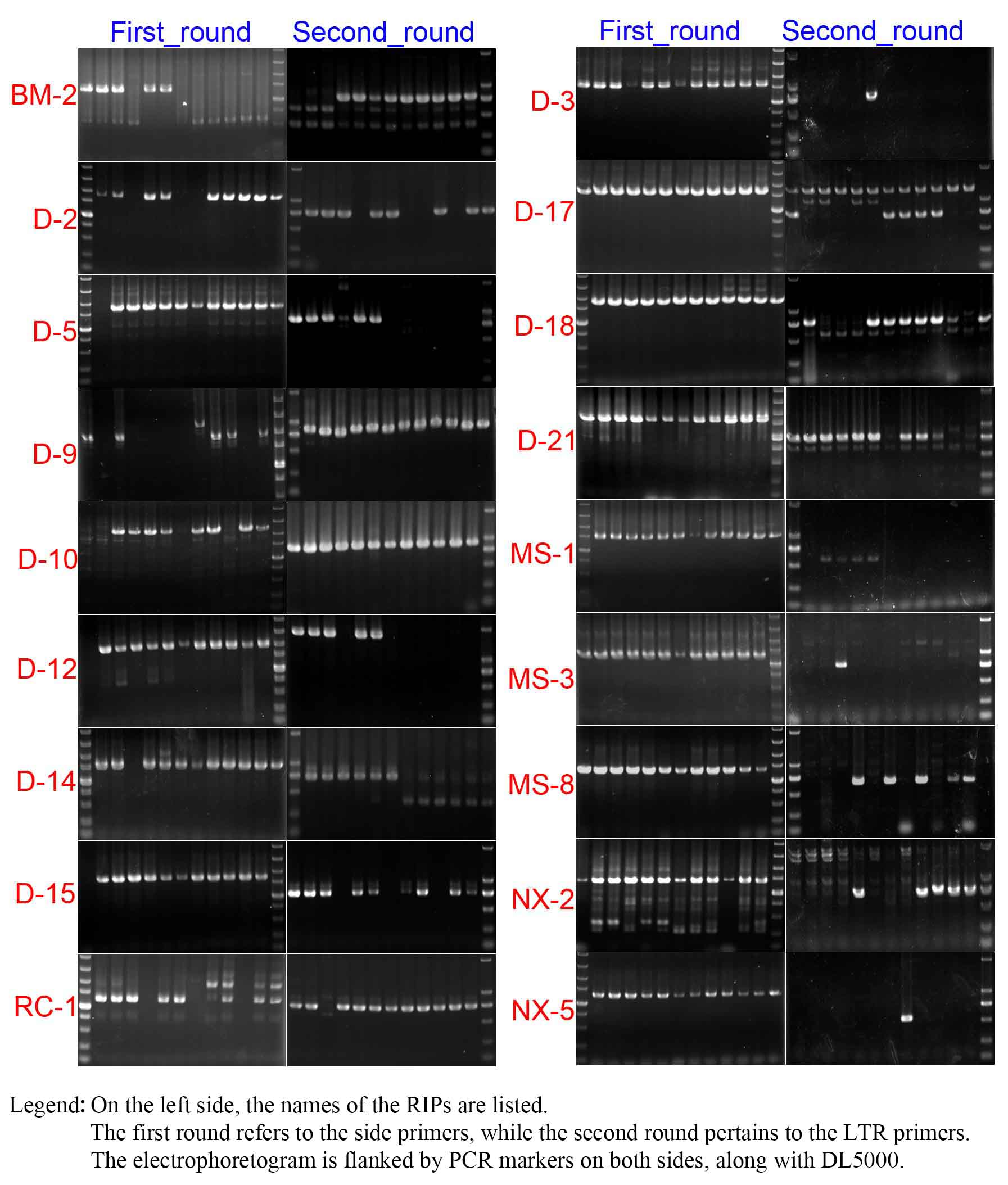

Supplement: Supplementary file 1 [file animals-14-00621-s001.zip › Supplementary Figure S3. Polymorphism identification of Fl-ERV in 12 pig breeds.jpg]

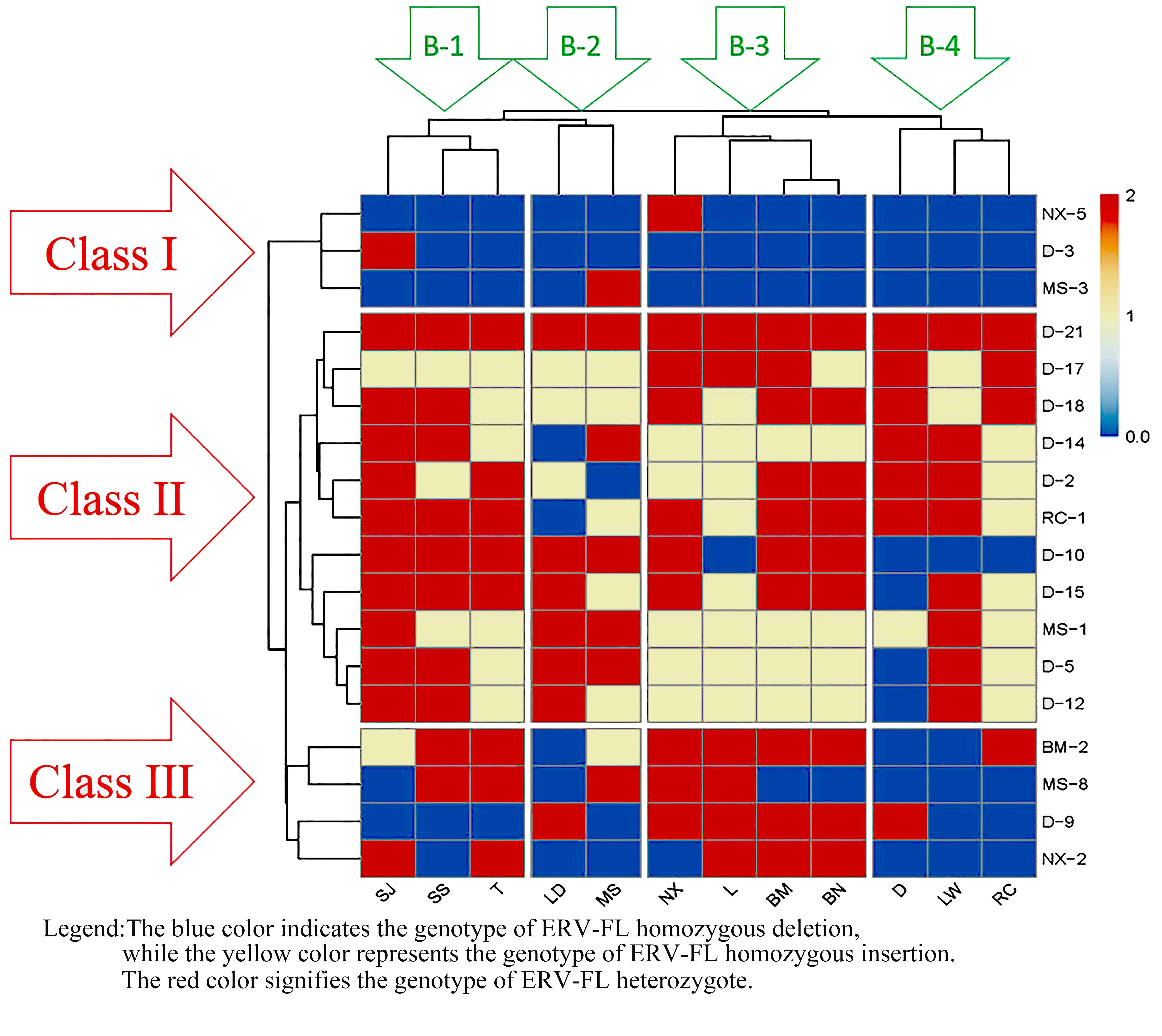

Supplement: Supplementary file 1 [file animals-14-00621-s001.zip › Supplementary Figure S4. Polymorphic heat maps of Fl-ERV in 12 varieties.jpg]

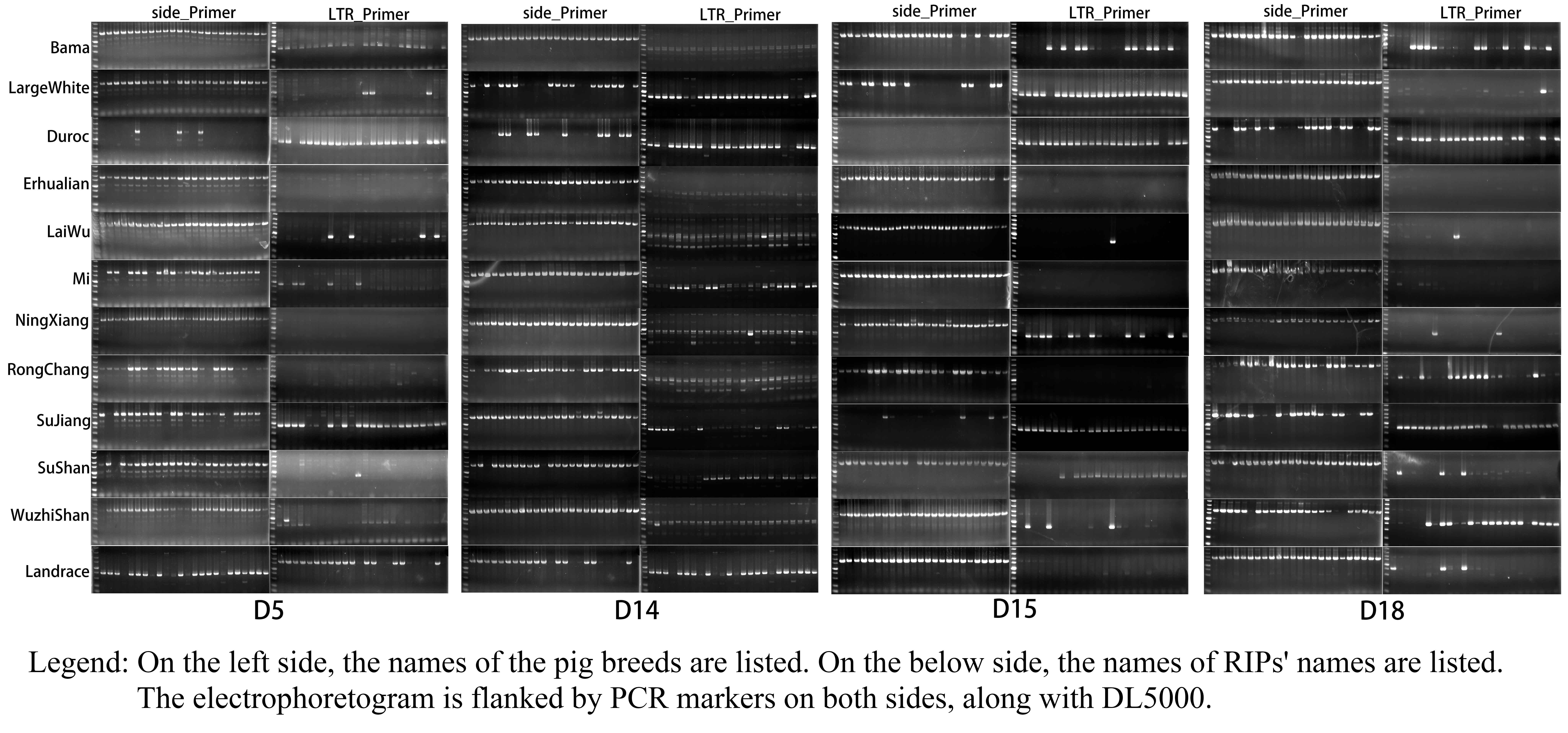

Supplement: Supplementary file 1 [file animals-14-00621-s001.zip › Supplementary Figure S5. Identification of 5 Fl-ERVs in 12 pig breeds.jpg]

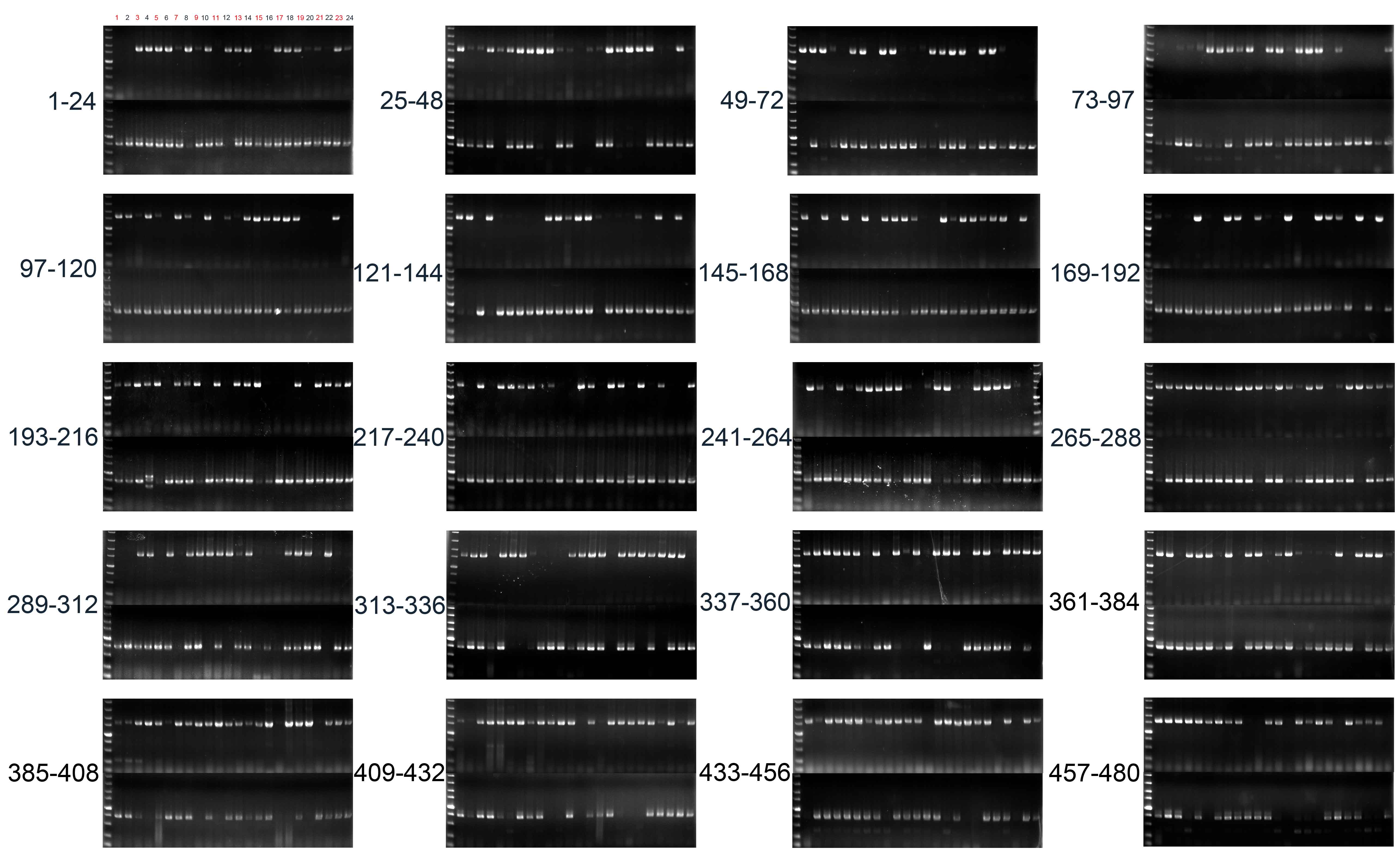

Supplement: Supplementary file 1 [file animals-14-00621-s001.zip › Supplementary Figure S6. Polymorphism identification of ERV-D14 locus in 480 large white pigs.jpg]

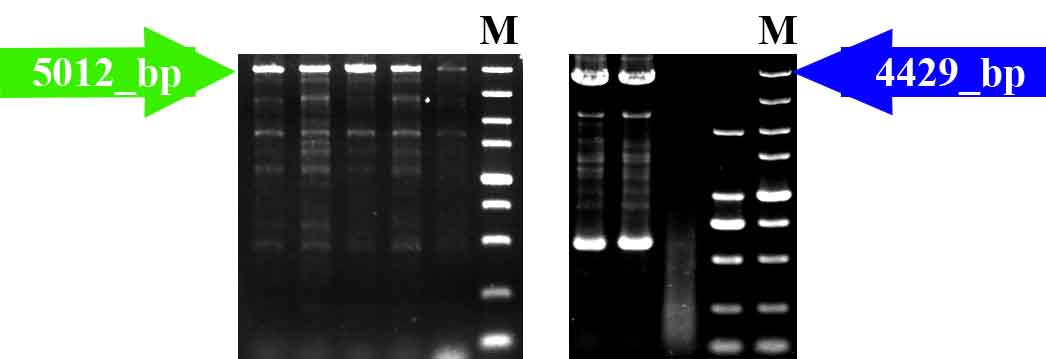

Supplement: Supplementary file 1 [file animals-14-00621-s001.zip › Supplementary Figure S8. Amplification of ERV-D14 in the intercalated Sushan pig genome.jpg]
